# siRNA-mediated therapeutic approaches improve acute kidney injury and limit its worsening

**DOI:** 10.3389/fphar.2025.1697460

**Published:** 2025-11-06

**Authors:** Xijian Wang, Xinzhong Huang, Bin Yang

**Affiliations:** 1 Nantong-Leicester Joint Institute of Kidney Science, Affiliated Hospital of Nantong University, Medical School of Nantong University, Nantong, China; 2 Department of Nephrology, Affiliated Hospital of Nantong University, Nantong, China; 3 Renal Research Group, Cardiovascular Sciences, University of Leicester, University Hospitals of Leicester, Leicester, United Kingdom

**Keywords:** small interfering RNA, acute kidney injury, chemical modifications, delivery systems, therapeutic targets

## Abstract

Acute kidney injury (AKI) is a critical clinical condition, with high morbidity and mortality globally, and also often worsening or progresses to chronic kidney disease (CKD). Despite advances in supportive and replacement therapy, specific interventions remain limited, in term of targeting a molecule (s) involved in the mechanism underlying AKI and its chronic progression. Recent developments in the technology of RNA interference (RNAi), particularly small interfering RNA (siRNA), offer promising avenues for the specific modulation of genes involved in AKI. This review highlights the potential of siRNA-mediated gene therapy to mitigate AKI and prevent its worsening. Here, the properties and advantages of siRNA agents were addressed. More importantly, the existing research on siRNA chemical modifications and delivery systems enabled specific and precise treatments for AKI, while some extensively studied therapeutic approaches were addressed. Furthermore, the challenges and future prospects of siRNA-based drug development for AKI were discussed, with aims to nourish re-searchers and clinicians alike, and promote establishing efficient organ/cell targeted delivery systems and accelerate potential clinical applications.

## 1 Introduction

Acute kidney injury (AKI) is a severe clinical syndrome characterized by a rapid and significant reduction in kidney function (Kellum et al., 2021), which has become a serious public health problem. Until now, there are no effective medications available for the prevention or treatment of AKI, which often worsening, and even progresses to chronic kidney disease (CKD) (Kork et al., 2015). Therefore, the development of cause-specific treatments for AKI is necessary and imperative. In the past decade, precision medicine has become a highly regarded hot topic in the healthcare field, facilitated by advancements in the technology of genome sequencing and bioinformatics (Bahcall, 2015; Jameson and Longo, 2015). One of promising avenues within the range of precision medicine is RNA interference (RNAi) therapy that showed a great potential for different diseases (Dammes and Peer, 2020; Wang et al., 2020). RNAi therapeutics using small interfering RNA (siRNA), microRNA (miRNA) and short hairpin RNA (shRNA) have been applied for various ailments, while this interview will focus on siRNA for the kidney injury.

siRNA is a nucleic acid-based therapeutic agent that mediates selective silencing of disease-associated genes via sequence-specific binding, offering considerable potential for the treatment of various diseases and representing a promising tool for precision medicine (Hu et al., 2020; Yoon et al., 2022). Theoretically, siRNA can be designed to target virtually any genetic locus of interest, affording advantages such as shorter development timelines, broader therapeutic applicability, and greater versatility compared to conventional small molecule drugs or antibodies (Dong et al., 2019). Furthermore, advancements in nucleic acid synthesis technologies have facilitated the precise, efficient, and cost-effective production of siRNA molecules (Bumcrot et al., 2006; Beaucage, 2008).

**FIGURE 1:**
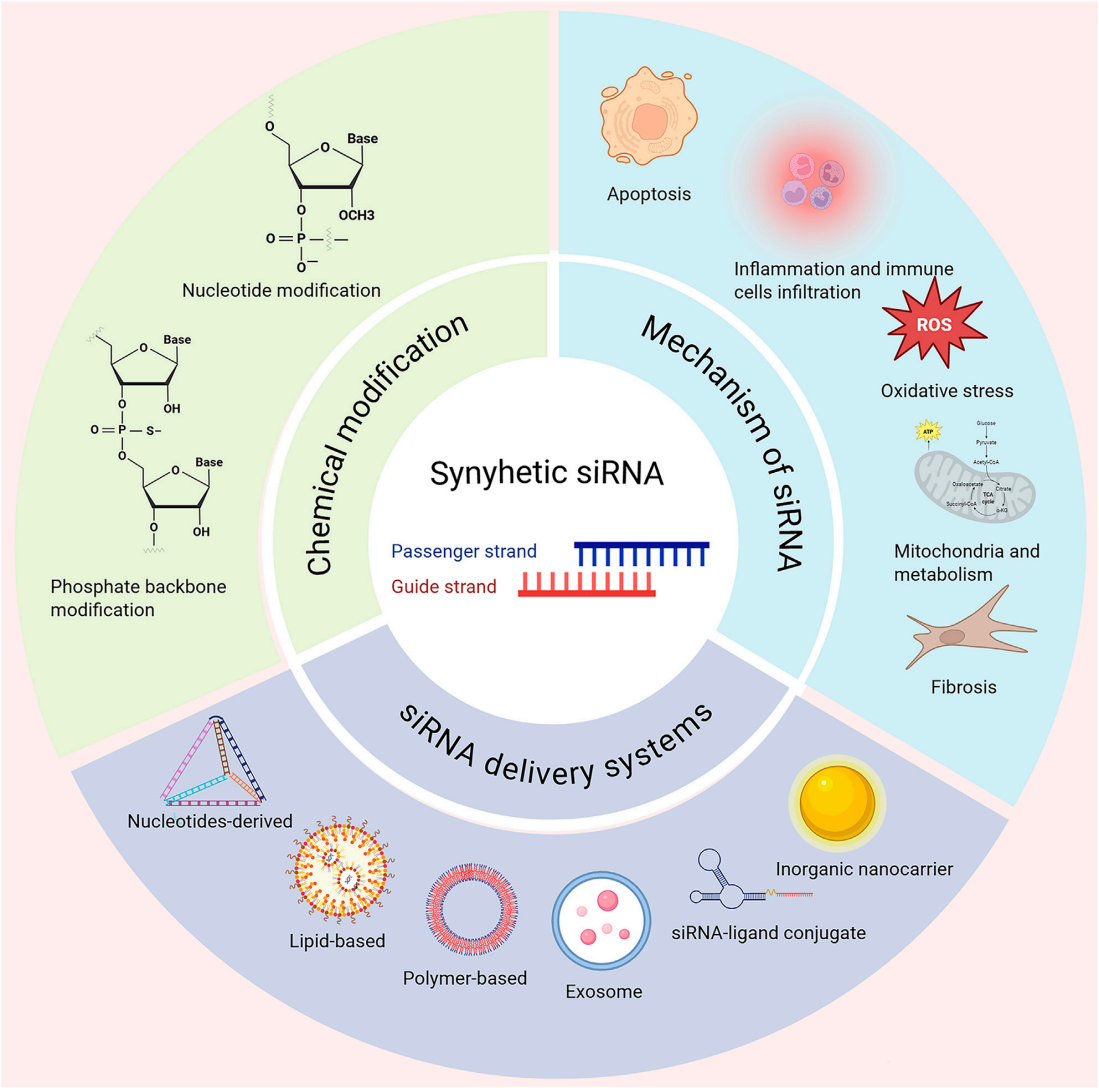
Chemical modifications, delivery systems and associated mechanisms are shown. siRNA through silencing target gene offers a highly precise therapeutic strategy for different disease settings including acute kidney injury (AKI). Chemically modified siRNAs include nucleotide and phosphorothioate backbone modifications. Advanced delivery systems using nucleotides-derived, lipid or polymer-based carriers, exosome, siRNA-ligand conjugate and inorganic nanocarrier. Once delivered, the siRNA modulates key genes in disease-related pathways, offering targeted therapeutic intervention. Created in BioRender. Wang, X. (2025) https://BioRender.com/f5jxsfc.

Notably, siRNA exhibits significant therapeutic potential for AKI (Thai et al., 2020; Fei et al., 2024; Gu X-R. et al., 2024). This review provides an overview of siRNA-based therapeutics including the chemical modification of siRNA to enhance stability and reduce immunogenicity in drug development, the latest development of siRNA delivery systems to improve organ such as kidney targeting and cellular uptake, and the mechanism of siRNA-based RNAi in the pathogenesis of diseases, for instance renal injury including inflammation, oxidative stress, mitochondrial dysfunction and cell death (apoptosis), following repair or fibrosis (Figure 1), with focuses on AKI and its chronic progression. siRNA therapeutics have been approved for marketing or currently undergoing clinical trials are also discussed. Finally, the critical challenges in the development of siRNA therapeutics and its prospects for clinical application in AKI are explored.

## 2 siRNA-mediated RNAi

### 2.1 Mechanism of siRNA action

**FIGURE 2:**
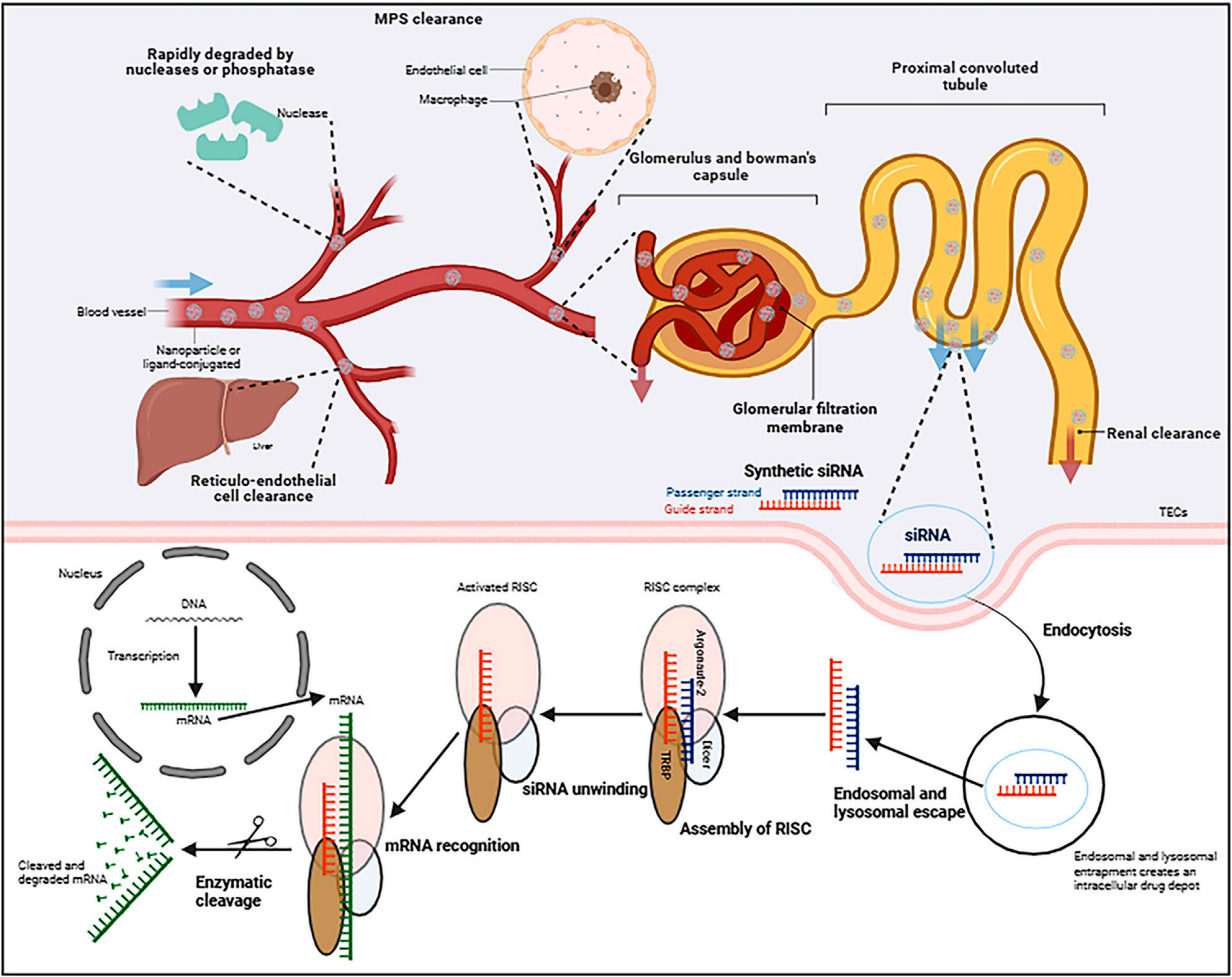
Schematic diagram of mechanisms of synthesizing small interfering RNA (siRNA) silencing a desired gene in kidney cells. Following systemic circulation, successful renal delivery siRNA requires against serum enzymes and immune system, then overcoming different barriers before entering target renal cells. In the cytoplasm of cells, the siRNA incorporates into RISC, the guide strand directs AGO2-mediated mRNA cleavage, resulting in target gene silencing. RISC, RNA-induced silencing complex; AGO2: argonaute 2. Created in BioRender. Wang, X. (2025) https://BioRender.com/k981uyo.

siRNA is a short form of 21–24 base pair nucleic acids duplexes that can induce RNAi in human cells, but faces multiple systemic challenges. If siRNA was administrated intravenously for instance, to achieve successful kidney delivery requires escaping serum nuclease degeneration and reticula-endothelial cell phagocytosis, overcoming the glomerular filtration barrier and tubular tight junctions, then entering cells such as tubular epithelial cells (TECs). Mechanistically, the native dsRNA is recognized and processed by the endoribonuclease Dicer, and then cleaved into small molecules of approximately 20 nucleotides, which is similar to chemically synthesized siRNA. These fragments are then loaded onto an RNA-induced silencing complex (RISC) to trigger RNA recognition. Within the RISC, the two strands of dsRNA are referred to as the guide and passenger strand, respectively. The argonaute 2 (AGO2) protein degrades the passenger siRNA strand, whereas the guide siRNA strand directly binds to the target mRNA through base pairing, causing AGO2-mediated cleavage or degradation. Consequently, the synthesis of matching protein is significantly inhibited (Figure 2) (Kanasty et al., 2013; Lim et al., 2022).

### 2.2 Advantages of siRNA therapeutics

siRNA therapeutics have more developmental prospects due to their unique advantages. Unlike conventional gene therapies, siRNA mediates sequence-specific gene silencing through RISC without genomic integration, thereby minimizing the risk of host gene mutations. Furthermore, siRNA efficiently performs its function through base pairing, and even with only a few fragments, it can induce significant gene silencing effects in cells. The small molecule of 21–24 nucleotides can precisely recognize target gene with high specificity, with modern bioinformatics algorithms and chemical modifications further minimizing off-target effects. Importantly, siRNA, designed based on the mRNA sequence of target gene, has multifunctionality, and can theoretically target any gene and treat any disease, due to advances in molecular biology and whole genome sequencing (Hu et al., 2020; Singh et al., 2018). These combined attributes to exceptional safety, high potency, precise specificity, and broad therapeutic applicability, which position siRNA as a transformative modality in precision medicine.

### 2.3 Updated development of siRNA therapeutics

Recent advances in RNAi therapeutics have positioned siRNA as a leading modality in drug development, with numerous candidates progressing through preclinical and clinical trials (Singh et al., 2018). To optimize siRNA therapy, it is necessary to determine whether local or systemic knockdown target gene is required, in order to improve delivery efficiency and reduce side effects. In addition, the same gene may have different impacts on different types of cells. For example, p53 in proximal tubular cells (PTCs) promotes AKI, while p53 in other tubular cells does not (Zhang et al., 2014). Furthermore, siRNA that induces apoptosis should be directly delivered into tumor cells rather than the surrounding normal cells. Therefore, cell-specific delivery of siRNA is crucial.

However, developing cell-targeted siRNA therapies has proven highly challenging. This is largely attributed to the cell diversity and delivery route complexity, as well as numerous encountered biological barriers (both extra and intra cellular), which hinder their safe and effective delivery to the interior of target cells (Figure 1). To address these challenges, the implementation of chemical modifications to siRNA molecules and/or the development of efficient delivery systems are critical for improving their bioavailability within target tissues and enhancing therapeutic outcomes (Van de Vyver et al., 2022; Ahn et al., 2023).

Extensive chemical modifications have been employed to optimize siRNA characteristics, such as increasing stability, minimizing off-target effects, and lowering immunogenic responses (Corey, 2007; Cho et al., 2009; Khvorova and Watts, 2017). Concurrently, advanced delivery platforms are vital for cell-specific targeting, significantly increasing the *in vivo* bioavailability of siRNA therapeutics. A variety of delivery strategies have been established and widely applied for siRNA transport, including lipid (Wan et al., 2014; Song et al., 2019), polymer (Neuberg and Kichler, 2014; Videira et al., 2014) or conjugate-based delivery systems (Thangamani et al., 2021; Ma et al., 2022).

Currently, many pharmaceutical companies have several siRNA therapeutics in their pipelines (Ghosh et al., 2020; Hodgson, 2020; Uludağ et al., 2020). Number of siRNA therapeutic drugs have already been approved by the US Food and Drug Administration (FDA, Table 1). However, the liver was the target organ for all of siRNA to exert their effects. Delivering siRNA to extrahepatic tissues represents the next major frontier in oligonucleotide therapeutics. As of January 2025, approximately 20 additional siRNA-based therapeutics have advanced to Phase 2 clinical trials or beyond globally. These candidates span a diverse spectrum of therapeutic areas, encompassing not only rare and genetic disorders but also extending to more common diseases (Table 1).

**TABLE 1:** siRNA therapeutics approved for marketing or in late-stage clinical trials.

Name	Phase and NCT NO.	Gene	Delivery technology	Disease	Administration
Patisiran	Approved	TTR	L NPs	hATTR	i.v.
Givosiran	Approved	ALAS1	GalNAc-siRNA conjugate	AHP	s.c.
Lumasiran	Approved	HAO1	GalNAc-siRNA conjugate	PH1	s.c.
Inclisiran	Approved	PCSK9	GalNAc-siRNA conjugate	Hypercholesterolemia	s.c.
Vutrisiran	Approved	TTR	GalNAc-siRNA conjugate	Polyneuropathy of hATTR amyloidosis	s.c.
Rivfloza	Approved	LDHA	GalXC™ RNAi platform	PH1	s.c.
Cemdisiran, ALN-CC5	Phase 2/3, NCT03841448 NCT05070858 NCT06479863 NCT05744921 NCT06541704 NCT05133531	CC5	GalNAc-siRNA conjugate	IgA nephropathy, PNH, geographic atrophy, sporadic inclusion body myositis, generalized myasthenia gravis	s.c.
ADX-038	Phase 1/2, NCT05876312	IL-6R	L NPs	PNH	i.v.
STP705	Phase 2, NCT04669808 NCT04844983 NCT04844840 NCT05196373	COX-2, TGF-β1	PNPs	BCC, intraepidermal SCC, skin SCC *in situ*, keloid	s.c., i.d., ita
SLN360	Phase 2, NCT05537571	APOA1, Lp (a)	GalNAc-siRNA conjugate	Cardiovascular diseases, atherosclerosis	s.c.
Olpasiran, AMG 890, ARO-LPA	Phase 2, NCT04270760 Phase 3, NCT05581303	APOA1, Lp (a)	GalNAc-siRNA conjugate	Cardiovascular disease, atherosclerotic cardiovascular disease	s.c.
ARO-APOC3	Phase 3, NCT05089084	APOC3	GalNAc-siRNA conjugate	Type I hyperlipoproteinemia, hypertriglyceridemia, congenital lipid metabolism disorders	s.c.
ALN-KHK	Phase 1/2, NCT05761301	Ketohexokinase	L NPs	Obesity, Obesity with type 2 diabetes	s.c.
Zilebesiran	Phase 1/2, NCT06423352 Phase 2, NCT04936035 NCT05103332	AGT	GalNAc-siRNA conjugate	Hypertension	s.c.
SLN124	Phase 1/2, NCT05499013	TMPRSS6	GalNAc-siRNA conjugate	Polycythemia vera	s.c.
Tivanisiran, SYL1001	Phase 3, NCT03108664 NCT04819269 NCT05310422	TRPV1	None (Unmodified, carrier-free)	Dry eye disease, Sjögren’s syndrome	o.a.
SYL1801	Phase 2, NCT05637255	NRARP	None	Wet macular degeneration, neovascular age-related macular degeneration, macular degeneration	o.a.
ALN-HSD	Phase 2, NCT05519475	HSD17B13	GalNAc-siRNA conjugate	Non-alcoholic steatohepatitis	s.c.
OLX10010	Phase 2, NCT04877756	CTGF	Cell-penetrating asymmetric siRNA	Hypertrophic scarring	i.d.
siG12D-LODER	Phase 2, NCT01676259	KRAS	LODER®	Pancreatic ductal adenocarcinoma	ita.
AOC 1020	Phase 1/2, NCT05747924	DUX4	Antibody-siRNA conjugate	Facioscapulohumeral Muscular Dystrophy	i.v.
Xalnesiran	Phase 2, NCT04225715	HBV gene	GalNAc-siRNA conjugate	HBV	s.c.
Imdusiran	Phase 2, NCT06154278	HBV gene	L NPs	HBV	s.c.
RBD1016	Phase 2, NCT05961098	HBV gene	GalNAc-siRNA conjugate	HBV	s.c.

TTR, transthyretin; L NPs, lipid nanoparticles; hATTR, polyneuropathy of hereditary TTR-mediated amyloidosis; ALAS1, aminolevulinate synthase 1; GalNAc, N-acetylgalactosamine; AHP, acute hepatic porphyria; HAO1, hydroxy acid oxidase 1; PH1, primary hyperoxaluria type 1; PCSK9, proprotein convertase subtilisin/kexin type 9; LDHA, lactate dehydrogenase A; CC5, complement component 5; IL-6R, interleukin-6 receptor; PNH, paroxysmal nocturnal hemoglobinuria; BCC, basal cell carcinoma; SCC, squamous cell carcinoma; APOA1, apolipoprotein A1; Lp (a), lipoprotein (a); APOC3, apolipoprotein C-III; AGT, angioten-sinogen; TMPRSS6, transmembrane serine protease 6; TRPV1, transient receptor potential cation channel subfamily V member 1; NRARP, NOTCH, regulated ankyrin repeat protein; HSD17B13, hydroxysteroid 17-beta dehydrogenase 13; CTGF, connective tissue growth factor; KRAS, kirsten rat sarcoma viral oncogene homolog; LODER®, local drug eluting release; DUX4, double homeobox 4; HBV, hepatitis B virus; i.v., intravenous injection; s.c., subcutaneous injection; i.d., intradermal injection; i.ta., intratumoral ad-ministration; o.a., ophthalmic administration.

Teprasiran was once highly anticipated as a drug for treating AKI induced by ischemia reperfusion (IR) injury. IR injury promotes cell injury and death via the transcription factor p53, which activates multiple signaling pathways. Teprasiran targets p53, inhibiting its role in growth arrest and apoptosis following IR injury (Thielmann et al., 2021). The phase 2 trial has shown that the incidence, severity, and duration of early AKI in high-risk patients undergoing cardiac surgery were significantly reduced after the administration of Teprasiran (ClinicalTrials.gov: NCT02610283). Furthermore, a phase 3 study was designed on the basis of these findings to access 90-day primary outcome of major adverse kidney events (ClinicalTrials.gov: NCT03510897). The designed primary endpoint at day 90 was completed, but 1 year follow up was terminated early due to the results not meeting the efficacy of outcome at day 90. The observed results in the Phase III study not meeting efficacy may be attributed to several factors. Firstly, the role of p53 across different renal cell types and stages of AKI remains incompletely defined. Key signaling pathways regulating p53 activation and its principal downstream effectors in AKI have not been fully elucidated. This limited understanding of target biology likely constitutes a major constraint on therapeutic efficacy. Secondly, as a systemically administered siRNA agent, the renal enrichment and retention of Teprasiran, particularly in PTCs, are critical to its effect. Current delivery systems may be inadequate to achieve and sustain sufficient drug concentrations within target cells during the critical injury window to fully suppress p53. Moreover, cardiac surgery patients often present with pre-existing renal impairment or intraoperative hemodynamic instability, which can further perturb drug distribution and clearance. These factors make it challenging for a single preoperative dose to achieve consistent and robust target silencing across all individuals. Therefore, future research should aim to clarify the cell type specific functions and regulatory mechanisms of p53 during AKI and subsequent renal repair, and to define its precise role at different disease stages. Such insights would facilitate the development of pharmacological modulators to prevent AKI or halt its progression to CKD. In terms of dosing strategy, there is a need to develop intelligent, injury-responsive postoperative regimens. These could include repeated or sustained administration within a specific therapeutic window guided by injury biomarkers, replacing the current single preoperative prophylactic approach. This strategy, in addition, could more precisely target p53-driven injury peaks, while minimize interference with physiological repair processes.

To our knowledge, there are currently no siRNA drugs for treating AKI and its chronic progression in the clinical research stage. However, recent clinical studies of siRNA drugs (Table 1), in particular GalNAc-siRNA conjugates treating IgA nephropathy, have strengthened our confidence. Our group has been extensively studying siRNA therapy for AKI in different animal models. Naked caspase-3 siRNA and TGF-β siRNA-loaded nanoparticles (NPs) have demonstrated outstanding renal protective effects on IR-induced AKI (Fei et al., 2024; Yang et al., 2014; Wu et al., 2020). Furthermore, the target cell delivery of siRNA-based reagents, caspase-3 siRNA conjugated with helix B surface pep-tide (HBSP) or cyclic HBSP (CHBP) conjugates, was also validated in isolated porcine kidneys subjected to IR injury and perfused by an isolated organ perfusion system (Black et al., 2022). This pilot study revealed these conjugates improved renal blood flow and urine output over a period of 4 h perfusion.

## 3 siRNA delivery systems

As mentioned above, siRNA can effectively inhibit mRNA transcription and subsequent protein translation by binding to a specific sequence of target gene. However, before siRNAs reached target genes there are numerous obstacles to be overcome and also off-target impacts to be avoided, with some examples here: (1) Unmodified naked siRNAs are unstable and prone to rapid enzymatic and nonenzymatic degradation, with a half-life in the bloodstream typically <5 min (Gao et al., 2009). (2) Immune system phagocytic clearance, as well as renal clearance. (3) Limited cellular uptake for most cell types, including renal cells, as the strong electronegative and macromolecular properties of siRNA make it difficult to penetrate the cell membrane into the cytoplasm. (4) Off-target effects including immune stimulation induced by siRNA, in particular using viral delivery vehicles (Gu X-R. et al., 2024). Therefore, the development of efficient and safe delivery strategies is essential to advance siRNA toward clinical application in humans.

Delivery systems are broadly categorized into viral and non-viral approaches. Viral vectors are recognized for their high delivery efficiency of nucleic acids. However, their clinical application is constrained by several limitations. These include a restricted nucleic acid cargo capacity of up to 5 kb, potential immunogenicity and toxicity concerns, and the risk of insertional mutagenesis (Kotterman and Schaffer, 2014; David and Doherty, 2017). The detailed description of viral vectors is not within the scope of this review, as there are numerous comprehensive reviews on this topic. Non-viral strategies can be distinguished between covalent conjugates and various nanoparticle-based delivery systems. The latter include various inorganic, lipid, polymeric, or other carriers mediating siRNA adsorption or encapsulation, and improving siRNA protection, cellular uptake and intracellular processing to the subcellular site of action (Ali Zaidi et al., 2023).

siRNA delivery systems for kidneys, in essence, should fulfill at least the following requirements: (1) maintaining siRNA stability through efficient endosomal/lysosomal escape mechanisms; (2) minimizing immunogenicity while evading immune recognition; (3) optimizing renal targeting efficiency and promoting cellular internalization; and (4) demonstrating excellent biocompatibility with minimal toxicity. Only when these conditions were met, the efficient delivery of siRNA drugs to kidneys can be achieved. In fact, the kidney may be an ideal target organ for siRNA therapy, as it has anatomical and physiological characteristics including rapid and vast blood flow, glomerular filtration and subsequent tubular absorption. PTCs are the main site for rapid and extensive endocytic uptake of siRNA after glomerular filtration (Yang et al., 2015a). This phenomenon has been demonstrated in preclinical studies. In a cisplatin-induced AKI model, naked synthetic siRNA targeting p53 injected intravenously 4 h after IR injury reduced upregulated p53 expression, protecting both PTCs and kidney function (Molitoris et al., 2009). In another study, Thompson et al. reported the pharmacokinetic properties of the synthetic siRNA (I5NP) inhibiting expression of the pro-apoptotic protein p53 following intravenous administration in rodents and nonhuman primates. I5NP was found to be filtered readily from the blood into the glomerular space, and then rapidly re-absorbed by PTCs (Thompson et al., 2012). Furthermore, advanced delivery strategies have been developed to enhance siRNA targeting to injured PTCs, including the use of inulin-conjugated C-X-C motif chemokine receptor 4 (CXCR4) ligand-modified polyplexes. This approach has demonstrated therapeutic efficacy in cisplatin-induced AKI, highlighting the promise of siRNA-based interventions for renal diseases (Jogdeo et al., 2024).

## 4 Technology platforms for siRNA therapeutics in AKI

More recently, the development of targeted delivery siRNA therapeutics for AKI is in full swing, due to several advances in siRNA chemistry, as well as the development of complementary delivery technologies. Various delivery systems have emerged as common strategies to facilitate the therapeutic application of siRNA drugs (Table 2).

**TABLE 2:** Recent advances in siRNA therapeutics for AKI.

Delivery system	Composition	AKI type	Gene	Outcome	Advantage	References
Polymer-based nanocarriers	Chitosan modified with C-CS	IRI	p53	Significantly ameliorated renal dysfunction through suppression of inflammatory responses and inhibition of apoptosis.	Favorable targeting and transfection efficiency	Tang et al. (2022a)
	Chitosan	Cisplatin	p53, PKCδ, γGT	Prevented cisplatin-induced nephrotoxicity via silencing of key pathogenic proteins.	Favorable targeting and transfection efficiency	Aydin et al. (2022)
	PLGA/CS/HA-KTP	Contrast	Arg-2	Markedly mitigated renal injury by alleviating oxidative stress, restoring mitochondrial function, and reducing apoptosis.	Biocompatibility, high encapsulation efficiency, favorable transfection efficiency	Gu et al. (2024b)
	PMVs@PLGA complexes	IRI, UUO	TGF-β	Potently attenuated renal fibrosis through targeting of the TGF-β1/Smad3 axis and suppression of inflammatory processes	Biosafe, favorable targeting and transfection efficiency	Fei et al. (2024)
	G5-FA	IRI	PHD2	Significantly improved kidney functions, kidney injury markers, and morphologic damage.	Favorable targeting and transfection efficiency	Xie et al. (2021)
	Inulin-CPTA	Cisplatin	p53	Markedly reduced tubular cell death, renal injury, inflammation, and overall improved renal function.	Excellent targeting	Jogdeo et al. (2024)
	Polyplexes	IRI, cisplatin	p53, CXCR4	Effectively reduced kidney damage and inhibited apoptosis.	Favorable targeting and transfection efficiency	Tang et al. (2022b)
Lipid-based nanoparticles	anti-VCAM-1 SAINT-O-Somes	TNFα, LPS	p65	Potently suppressed endothelial inflammatory response.	Favorable targeting and transfection efficiency, biocompatible, lower toxicity	Kowalski et al. (2014)
Nucleotides derived nanostructure	L-sTd	Folic acid	p53	Effectively attenuated apoptosis, restored renal structural integrity, and mitigated functional deterioration.	Favorable targeting and transfection efficiency	Thai et al. (2020)
	TDN-3Chol	Cisplatin	p53	Significantly mitigated the incidence of apoptosis in renal histocyte.	Favorable targeting, lower toxicity	Li et al. (2024)
Extracellular vesicle platform	REVLTH	IRI, UUO	p65, Snai1	Significantly improved kidney injury by alleviating tubulointerstitial inflammation and fibrosis, and potently abrogated the transition to CKD.	Biosafe, biocompatible, nonimmunogenic	Tang et al. (2021)
siRNA-ligand Conjugate	HA-KTP/PSPD	Drugs	Arg-2	Enhanced mitochondrial autophagy, mitigate oxidative stress, and inhibit apoptosis, ultimately improving renal function.	Efficient siRNA condensation, high loading capacity, protection against degradation, PH responsive release	Gu et al. (2024a)
Inorganic nanocarriers	fCNT	Cisplatin	p53, Mep1b	fCNTs were first evaluated in nonhuman primates, demonstrating that targeted gene delivery effectively reduced renal injury, fibrosis, and immune infiltration.	Easy synthesis, biocompatible, lower toxicity	Alidori et al. (2016)

C-CS, chitosan modified with α-cyclam-p-toluic acid; IRI, ischemia reperfusion injury; PKC-δ, protein kinase C-δ; γ-GT, γ-glutamyl transferase; PLGA, poly(lactide-co-glycolide); HA-KTP, hyaluronan kidney-targeting peptide; Arg-2:Arginase-2; UUO, unilateral ureteral obstruction; PMVs, platelet membranes; G5-FA, folic ac-id-decorated poly-amidoamine dendrimer generation 5; PHD2, prolyl hydroxylase domain protein 2; CPTA, α-cyclam-p-toluic acid; CXCR4, C-X-C Chemokine Receptor Type 4; TNFα, tumor necrosis factor α; LPS, lipopolysaccharide; VCAM-1, vascular cell adhesion protein 1; L-sTd, L-small DNA, tetrahedrons; TDN-3Chol, tetrahedral DNA, nanostructure modified with three cholesterol molecules; PSPD, polymeric spermidine; fCNT, ammonia-functionalized single-walled carbon nanotube.

### 4.1 Chemical modification of siRNA

Although simple chemical modifications are not sufficient to act as delivery vectors for siRNA, they are pivotal in augmenting the intrinsic characteristics of siRNAs. These enhancements include notable gains in serum stability, the ability to evade immune recognition, and more efficient incorporation into the RISC (Whitehead et al., 2009; Czech et al., 2011). Owing to their importance, a solid foundation in chemical modification strategies is necessary prior to examining delivery platforms. In general, such modifications are divided into two main categories: nucleotide alterations and phosphate backbone adjustments (Ali Zaidi et al., 2023).

Drawing on the native architecture of ribonucleotides, chemical changes can be introduced at the nucleobase, sugar residue, or nucleoside level. Empirical studies indicate that the replacement of uridine with 2′-O-methyluridine boosts siRNA stability and minimizes off-target silencin (Dong et al., 2019). Other base substitutions comprise the use of 2′-deoxy-2′-fluoro cytidine in place of cytidine (Akabane-Nakata et al., 2021), N6-methyladenosine for adenosine (Rydzik et al., 2021), 2′-O-methyl guanosine for guanosine (Jackson and Linsley, 2010), and 2-thiouridine for uridine (Anderson et al., 2011). Regarding the potential impact of these modifications on RISC loading efficiency, sugar modifications in siRNA design have been commonly adopted including the exchange of ribose with either 2′-O-methyl ribose or 2′-fluoro ribose. These alterations, prevalent in commercial siRNA formulations, contribute to improved serum persistence and lower immunogenicity (Roberts et al., 2020). Another sugar modification, involving locked nucleic acid *in lieu* of ribose, has also been reported to increase siRNA stability (Kanasty et al., 2013), which has been applied extensively in our own research (Yang et al., 2014; Wu et al., 2020; Yang et al., 2011). However, its unique conformational properties may present synthetic challenges and increase production cost. Additionally, the attachment of a 5′-triphosphate cap is among the most prevalent nucleoside modifications, which is known to augment siRNA function by promoting RISC loading (Selvam et al., 2017). Nevertheless, the *in vivo* metabolic kinetics of this modification still need to be evaluated in specific application contexts.

To enhance resistance against nuclease-mediated degradation, the phosphodiester backbone of siRNA can be chemically altered through the introduction of phosphonate-based linkages. A widely utilized approach involves phosphorothioate (PS) modification, wherein a sulfur atom replaces one of the non-bridging oxygen atoms in the phosphodiester bond, resulting in a negatively charged phosphorothioate linkage. This modification enhances nuclease stability and promotes binding with plasma proteins, particularly albumin, potentially leading to prolonged circulation time. However, nonspecific protein binding is positively correlated with *in vivo* toxicity. Therefore, both the positioning and extent of phosphorothioate linkages are critical for their practical application (Ku et al., 2016). In the context of siRNA modification, Alnylam introduced two PS linkages at the first two nucleotides of the 5′-end of the sense strand, and two PS linkages at each of the 5′- and 3′-ends of the antisense strand (Hu et al., 2020). For clinical siRNA development, it is recommended to limit PS modifications to between 5% and 50% of total linkages, with the exact proportion adjusted according to the intended dose (Chernikov et al., 2019). Another strategy, phosphorodithioate (PS2) modification, involves the substitution of both non-bridging oxygen atoms with sulfur, leading to a chemically robust and nuclease-resistant conformation. PS2-modified siRNAs exhibit increased stability and biological efficacy, and have shown promising results in preclinical studies targeting cancer and viral diseases (Abeydeera et al., 2016). Additional phosphonate-derived modifications, the site-specific introduction of methylphosphonate and methoxypropylphosphonate modifications, has been employed to reduce drug-protein binding, thereby mitigating potential hepatotoxicity, since alkylphosphonate linkages are charge neutral, while the PS backbone is negatively charged, which changes the overall charge distribution of the molecule (Hu et al., 2020; Migawa et al., 2019). However, the electroneutral nature of these modifications may also influence the solubility and biodistribution of siRNA, presenting critical factors that must be carefully balanced during drug design.

Despite the availability of diverse modification strategies to refine siRNA properties, a careful appraisal of their unintended consequences is vital. Certain chemical alterations may impair siRNA functionality (Hall et al., 2004). Furthermore, as many modifying agents are foreign to human biology, it is imperative to conduct stringent safety assessments of chemically modified siRNAs in preclinical settings before any human therapeutic use.

### 4.2 siRNA-ligand conjugate

Directly conjugating ligands to siRNA represents a promising strategy for delivery. They play a crucial role in enhancing siRNA properties by improving cellular uptake and enabling cell type specific targeting. Various ligands, including carbohydrates, aptamers, peptides, and antibodies, have been covalently attached to siRNA to achieve these effects (Dong et al., 2019).

Peptides hold significant potential for enhancing nucleic acid delivery, due to their versatile and adaptable interactions with biomolecules and cells (Urandur and Sullivan, 2023). Building upon this potential, our research program has established a systematic framework for peptide-mediated siRNA delivery in renal therapeutics. We first established the renal protective effects of CASP3 siRNA in both isolated porcine kidney preservation (Yang et al., 2011) and auto transplantation models (Yang et al., 2014). Subsequent work revealed synergistic therapeutic effects when combining CASP3 siRNA with erythropoietin-derived peptides HBSP or CHBP (Wu et al., 2020), while later studies characterized the upregulated expression of innate repair receptors, also called EPOR/βcR, in injured kidney cells including renal epithelial cells (Wu et al., 2023). These studies paved a way of a potential new approach using siRNA conjugated with peptide HBSP or CHBP (a ligand of EPOR/βcR) guided by highly expressed EPOR/βcR on damaged cells including most vulnerable TECs to AKI to achieve delivering siRNA to desired target cells (Black et al., 2022). Notably, recent studies have further highlighted the therapeutic promise of siRNA-peptide conjugates for AKI intervention. KTP exhibits a specific affinity for megalin (Wischnjow et al., 2016), a membrane receptor highly expressed in renal TECs. The nanoparticles effectively delivered siRNA into kidney cells through the interaction between KTP and megalin, and facilitated successful endo/lysosomal escape, thus giving high selectivity and transfection efficiency. This delivery system demonstrated strong therapeutic efficacy in DI-AKI animal models (Gu X-R. et al., 2024), highlighting the particular promise of combining targeting ligands with nanoparticle delivery systems for renal siRNA applications.

siRNA conjugates demonstrate promising targeting capability and enhanced pharmacokinetic properties, making them an increasingly investigated delivery platform. However, their clinical translation faces critical challenges including limited gene transfer efficiency, high production costs, and safety concerns (Urandur and Sullivan, 2023; Yang and Bae, 2023). Overcoming these limitations will determine their therapeutic potential for AKI treatment.

### 4.3 Polymer-based nanocarriers

Polymer-based nanoparticles have emerged as versatile tools in medical biology research due to their unique properties, including biocompatibility, tunable size, and surface functionality (Su et al., 2021).

Chitosan is a naturally derived polymer obtained through the partial N-deacetylation of chitin, which is sourced from various natural origins (Ragelle et al., 2013). It is well-known that chitosan has biodegradability, biocompatibility, non-toxicity, and strong bio-retentive properties, which are attributed to its mucoadhesive nature (Cheung et al., 2015; Mohammed et al., 2017). This polymer readily forms complexes with polynucleotides, which carry a positive surface charge. The mucoadhesive property of chitosan allows it to mimic the mucus membrane, enabling the sustained release of therapeutic agents (TM et al., 2018). Low molecular weight chitosan, in particular, has gained prominence as a widely used polymeric carrier for the renal delivery of siRNA and other drugs, primarily due to its intrinsic cationic charge, which enhances its affinity for the negatively charged glomerular basement membrane (GBM). Additionally, the amino groups and glucosamine units in chitosan facilitate interactions with megalin receptors expressed on s (Yuan et al., 2007; Yuan et al., 2009; Yang et al., 2015b). In a study conducted by Tang et al., a novel siRNA delivery system using chitosan modified with α-cyclam-p-toluic acid (C-CS) for targeted AKI treatment. The C-CS polymer specifically targets cells overexpressing CXCR4, a receptor associated with AKI pathology, and showed superior kidney accumulation compared to its parent compound. C-CS/siRNA nanoparticles loaded with p53-targeting siRNA effectively reduced renal apoptosis, inflammation, and macrophage/neutrophil infiltration while improving kidney function in a mouse model of IR-AKI (Tang et al., 2022a). This study highlights a key innovation in AKI therapy by leveraging CXCR4 targeting for precise micro-environmental intervention, where the C-CS carrier not only enhances renal siRNA delivery, but also improves cellular uptake through receptor-mediated endocytosis. In addition, the work by Erkin et al. provided compelling evidence for the multi-target therapeutic potential of chitosan/siRNA nanoparticles. To prevent cisplatin damage, they designed chitosan/siRNA nanoparticles contained siRNA against cationic membrane transport (OCT1&2) and apoptosis related proteins (p53, protein kinase C-δ [PKC-δ], and γ-glutamyl transferase [γ-GT]). The siRNA nanoparticles effectively reduce serum creatinine and blood urea nitrogen levels in cisplatin-treated mice, significantly protecting kidney function. Treated mice showed decreased expression of p53, PKCδ, and γGT, along with histological evidence of preserved kidney PTCs. The protective effects correlated with the number of siRNA administrations (Aydin et al., 2022).

Inulin is a natural polysaccharide belonging to the fructan-type and has been explored as a carrier for siRNA delivery. It is biocompatible, hydrophilic, and easily amenable to chemical modifications due to its reactive functional groups, particularly hydroxyl groups. The highly flexible structure of inulin makes it a promising and versatile candidate for siRNA delivery applications (Moazzam et al., 2024). Jogdeo et al. synthesized inulin modified with α-cyclam-p-toluic acid (CPTA) to form a novel renal-targeted polymer, Inulin-CPTA (IC), which can selectively deliver siRNA to damaged kidneys. Self-assembled IC/siRNA nanoparticles (polyplexes) demonstrated rapid accumulation in the injured kidneys with selective CXCR4 and prolonged retention in injured renal tubules overexpressing the CXCR4 receptor. Systemically administered nanoparticles formulated using IC and siRNA against p53 selectively accumulated in the injured kidneys and potently silenced p53 expression, thereby improving kidney injury (Jogdeo et al., 2024). This CXCR4-targeted inulin platform demonstrates excellent renal injury-specific delivery and effective gene silencing, highlighting potential of inulin as a natural siRNA carrier for AKI therapy.

Poly(lactide-co-glycolide) (PLGA) is a commonly utilized biodegradable polymer that has been approvered by Food and Drug Administration and already being used in clinical settings (Wang et al., 2021). Known for its sustained release properties, biodegradability, and low toxicity, PLGA serves as an effective and versatile carrier for various therapeutic agents, including nucleotides and other small molecules (Panyam et al., 2002). Gu et al. developed the layer-by-layer assembled renal-targeting polymeric nanoparticles to efficiently deliver siRNA, knockdown Arginase-2 (Arg-2) expression in renal tubules, and evaluated the prevention of contrast-induced acute kidney injury (CI-AKI). These nanoparticles, composed of PLGA cores coated with cationic chitosan and anionic hyaluronan (HA), are further modified with a kidney-targeting peptide (KTP) to enhance renal accumulation. In CI-AKI models, the nanoparticles effectively reduce oxidative stress, restore mitochondrial function, and decrease apoptosis, demonstrating strong therapeutic potential for preventing CI-AKI (Gu XR. et al., 2024). This innovative layer-by-layer design combines the advantages of controlled release from PLGA with mucoadhesiveness from chitosan/HA and targeting specificity from KTP, representing a significant advancement in precision nanomedicine for renal diseases. Nevertheless, conventional PLGA nanoparticles still face significant biological barriers, particularly rapid clearance by the reticuloendothelial system (Figure 2) due to their inherent immunogenicity. To overcome these limitations, our research group has been committed to developing cell membrane coated NPs (CMC@NPs). Platelet membrane vesicles (PMVs) are a promising platform for designing NPs with enhanced immune-compatibility and the ability to specifically target injury sites. Recently, PMVs@TGF-β1-siRNA NP complexes were developed and their therapeutic potential was evaluated through both *in vitro* and *in vivo* studies. It was demonstrated that the kidneys were efficiently targeted by these PMVs@siRNA NPs in unilateral ureteral obstruction (UUO) and IR injury mouse models. In these models, TGF-β1 expression was significantly reduced, and kidney inflammation and fibrosis were alleviated by the PMVs@siRNA NP complexes through the inhibition of TGF-β1/Smad3 signaling pathway (Fei et al., 2024).

Dendrimers, in addition, are 3D polymers with a central core and branching structures, often based on amine-containing polymers. Dendrimers are recognized for their stability, low toxicity, and excellent water solubility, making them efficient carriers for siRNA delivery (Rehman et al., 2022). Xie et al. developed a folate receptor (FR)-targeted siRNA delivery system by conjugating folic acid (FA) to a generation 5 polyamidoamine dendrimer (G5-FA), which selectively binds to FR-expressing cells such as PTCs in the kidney. To evaluate its therapeutic potential, the researchers complexed G5-FA with siRNA targeting prolyl hydroxylase domain protein 2 (PHD2) and administered it via tail vein injection 24 h before renal ischemia. *In vivo* imaging confirmed predominant accumulation of the complex in the kidneys, with only minimal fluorescence detected in the liver. Consistent with this kidney-specific targeting, significant reductions in PHD2 mRNA and protein levels were observed exclusively in renal tissue, demonstrating the precision of carrier. Treatment with the G5-FA/PHD2 siRNA complex provided robust protection against IR injury, as evidenced by decreased serum creatinine and blood urea nitrogen levels, reduced kidney injury biomarkers, and attenuated histological damage (Xie et al., 2021). FA-modified dendrimers not only showcase excellent siRNA delivery capabilities but more importantly reveal substantial therapeutic promise for AKI intervention, particularly through their targeted action and preconditioning effects.

Other Polymer-based carrier materials have also been used to deliver siRNA to the kidney to provide potent treatments for AKI. A notable example is the cationic polymer carrier, PSPD, synthesized using spermidine (SPD) as a monomer for siRNA delivery, demonstrated efficient siRNA condensation, high loading capacity, protection against degradation, and pH responsive release (Gu X-R. et al., 2024). The intrinsic multifunctionality derived from SPD, alleviated drug-induced AKI (DI-AKI) by promoting mitochondrial autophagy, reducing oxidative stress, and inhibiting apoptosis. To improve renal targeting, a HA-KTP shell was applied to the PSPD/siRNA complex, slightly increasing its size and neutralizing its surface charge. This modification enhanced nanoparticle recognition by renal s, boosting targetability. The resulting HA-KTP/PSPD/siRNA system demonstrated strong therapeutic efficacy in DI-AKI animal models. Furthermore, Tang et al. reported the development and evaluation of a polymeric CXCR4 antagonist (PCX) as an siRNA carrier. The PCX/siRNA target p53 polyplexes demonstrated significant potential to enhance renal accumulation in AKI and effectively deliver therapeutic siRNA (Tang et al., 2022b). Both systems provide valuable insights for future AKI siRNA therapeutics development.

Polymer nanocarriers have emerged as a groundbreaking platform for siRNA delivery in AKI treatment, offering solutions to the longstanding challenges of nucleic acid stability, renal cell targeting, and intracellular delivery. These systems demonstrate remarkable potential to shift AKI management from supportive and replacement treatment to precise molecular intervention. However, key translational hurdles must be overcome before clinical implementation. Foremost among these is the establishment of robust, scalable manufacturing protocols to ensure batch-to-batch consistency of therapeutic-grade nanoparticles (Su et al., 2021). Additionally, the relatively low siRNA payload capacity of current formulations may limit their therapeutic index and clinical applicability (Dethe et al., 2022). Addressing these limitations through continued multidisciplinary research and rigorous pre-clinical validation will be essential to the full therapeutic potential of polymer nanocarriers for AKI patients.

### 4.4 Lipid-based nanostructure

Lipid-based nanoparticles, including liposomes, micelles, emulsions, and solid lipid nanoparticles, are extensively studied due to their favorable biocompatibility, biodegradable nature, low toxicity profile, and suitability for surface functionalization with targeting ligands and other bioactive molecules (Xin et al., 2017).

Liposomes represent one of the most promising nanocarrier systems for renal siRNA delivery, owing to their unique structural and physicochemical properties (Huang et al., 2024). These spherical vesicles, composed of a phospholipid bilayer enclosing an aqueous core, are versatile carriers for both hydrophobic and hydrophilic therapeutics due to their amphipathic nature (Wagner et al., 2017). Microvascular endothelial cells play a critical role in sepsis-induced inflammation and kidney injury, and are major contributors to the progression of CKD. Kowalski et al. developed a novel targeted liposome, SAINT-O-Somes, for delivering siRNAs to inflamed endothelial cells *in vivo*. These liposomes, based on the cationic amphiphile SAINT-C18 (1-methyl-4-(cis-9-dioleyl) methyl-pyridinium-chloride), were modified with antibodies against vascular cell adhesion protein 1 (VCAM-1) for specificity to inflamed endothelium. In tumor necrosis factor α (TNFα)-challenged mice, anti-VCAM-1 SAINT-O-Somes effectively targeted VCAM-1-expressing endothelial cells without causing liver or kidney toxicity. They successfully delivered siRNA to knock down vascular endothelial cadherin mRNA in inflamed renal microvasculature and attenuated the inflammatory response to lipopolysaccharide in kidney endothelial cells using nuclear factor kappa-B (NF-κB) p65-specific siRNA (Kowalski et al., 2014). While these cationic liposomes show promising targeting capabilities, it must be mentioned that in terms of biocompatibility and pharmacokinetics, anionic or neutral liposomes are usually superior to cationic liposomes due to the negative charge properties of biological membranes (Shang et al., 2024). This consideration highlights the need for careful design optimization when developing liposomal systems for renal siRNA delivery.

Lipid-based delivery systems hold great promise for overcoming the limitations of traditional renal therapies by enhancing drug efficacy, reducing systemic side effects, and enabling precision targeting in kidney diseases. However, key challenges remain in drug loading efficiency, particle size control, and formulation stability. Addressing these limitations through advanced lipid engineering will be crucial for clinical translation (Sumera et al., 2017).

### 4.5 Nucleotides derived nanostructure

Nucleotides have been widely used as fundamental building blocks for assembling various nanoparticles (Seeman, 2010). This is particularly exemplified by DNA nanostructures, which have undergone continuous development and refinement over the past 3 decades since their initial conception (Seeman, 2010).

NPs smaller than 100 nm can cross the kidney’s glomerular endothelial layer and be filtered through the GBM. The subsequent tubular reabsorption of these particles, contingent upon their uptake properties, leads to their accumulation in the kidney. Among various nanocarriers, DNA tetrahedrons emerge as particularly promising candidates due to the profile of optimal size, cellular internalization efficiency, and remarkable structural stability at physiological temperature (37 °C) (Thai et al., 2020). The researchers synthesized four small DNA tetrahedrons (sTds) through the self-assembly of sugar-backbone-modified oligonucleotides and evaluated their potential for kidney-specific distribution. Among them, the L-sTd demonstrated the highest localization efficiency in the kidney and was subsequently employed as a carrier for kidney-targeted delivery of siRNA target p53 mRNA (siP53). The siRNA-loaded L-sTd (siP53@L-sTd) was successfully delivered to the kidney, taken up by kidney cells, effectively downregulated p53 gene. This approach achieved a therapeutic effect on AKI at a remarkably low dose of 0.25 mg/kg per injection (Thai et al., 2020). The L-sTd exemplifies how precise nanostructure engineering enables ultra-low-dose efficacy by synergizing size-selective filtration with optimized cellular uptake. Moreover, Li et al. demonstrated that high-dimensional design combined with cholesterol modification can significantly extend the systemic circulation half-life of DNA nanostructures in mice. Specifically, a tetrahedral DNA nanostructure modified with three cholesterol molecules (TDN-3Chol) exhibited an extended circulation time and a strong preference for renal uptake. Using TDN-3Chol as a delivery platform, p53 siRNA was successfully transported into renal tubular cells, effectively mitigating CI-AKI (Li et al., 2024). By incorporating cholesterol moieties, the TDN nanostructure simultaneously achieves extended plasma half-life and preferential renal uptake, overcoming a key limitation in renal drug delivery.

DNA nanostructures are precisely engineered carriers that protect siRNA, enhance its stability, and improve pharmacokinetics for effective delivery. Despite their potential, these vectors in siRNA delivery face several drawbacks such as potential immune reactions, complex manufacturing, rapid clearance, and difficulty crossing biological barriers (Qu et al., 2022). These limitations may be addressed through improved designs including chemical modifications and hybrid systems, which is crucial for realizing the full potential of DNA nanostructures in siRNA therapeutics.

### 4.6 Extracellular vesicle platform

Exosomes are cell-derived nanovesicles that naturally transport biomolecules between cells, making them promising drug delivery vehicles (Valadi et al., 2007). Their innate biocompatibility and low immunogenicity allow them to efficiently deliver therapeutic cargo, such as siRNA, to target tissues. Additionally, exosomes can be engineered with surface modifications to further enhance their targeting precision and therapeutic potential (Tang TT. et al., 2022).

Stem cells have the unique ability to self-renew and facilitate tissue repair (Maqsood et al., 2020). Mesenchymal stromal cell (Cao et al., 2021) or embryonic stem cell (Yu et al., 2021) derived extracellular vesicles (EVs) have been shown to mitigate AKI and inhibit the progression of renal fibrosis in a murine model of renal IR injury. This highlights the therapeutic promise of EV-based strategies for kidney injury. In parallel, engineered exosomes have recently emerged as a promising delivery platform for RNA-based therapeutics. For example, studies have utilized EVs isolated from dendritic cells and fibroblasts, loaded with siRNAs through electroporation, to target Alzheimer’s disease (Alvarez-Erviti et al., 2011) and pancreatic cancer (Kamerkar et al., 2017), respectively. These examples illustrate the adaptability of engineered EVs for RNA delivery in diverse disease contexts. However, EVs derived from different cell sources exhibit variations in cargo composition, functional properties, and biodistribution patterns (Meng et al., 2020). Red blood cell-derived extracellular vesicles (REVs) are considered an ideal drug delivery vector. Firstly, red blood cells are inherently biosafe, biocompatible, and non-immunogenic, which constitutes a fundamental prerequisite for their potential use as drug carriers. Secondly, compared to other cell types, red blood cells are the most abundant cellular component in peripheral blood, facilitating their isolation. Furthermore, the absence of both nuclear and mitochondrial DNA in red blood cells further minimizes the risk of inducing unpredictable biological effects in recipient cells. Native REVs from type O human donors have been employed for the delivery of RNA therapeutics, including antisense oligonucleotides, Cas9 mRNA, and guide RNAs (Usman et al., 2018). Recently, Tang et al. provides further validation of the considerable clinical translation potential of REVs as a nanocarrier platform. Researchers ingeniously engineered Kim-1-binding LTH peptide-functionalized REVs containing dual siRNAs, enabling efficient targeting of injured kidney tubular cells highly expressing Kim-1. REVs delivering dual siRNAs specifically to kidney tubular cells silenced P65 and Snai1, and mitigated tubulointerstitial inflammation and fibrosis, thereby improving AKI caused by IR and UUO, and preventing progression to CKD (Tang et al., 2021).

EV platforms offer a transformative strategy for siRNA therapy, leveraging natural delivery mechanisms to achieve targeted, efficient, and safe gene silencing. However, the efficient loading of therapeutic cargoes into EVs remains one of the most significant challenges. Fortunately, several techniques for loading siRNA into EVs have been developed, which are broadly categorized into post-loading methods (e.g., incubation, electroporation, sonication, extrusion, and freeze/thaw cycle), pre-loading methods (e.g., cell transfection and co-incubation), and other loading methods (e.g., engineered parental cell and microfluidic synthesis of biomimetic lipid nanoparticles) (Meng et al., 2020). The loading efficiency varies considerably among these different methods, with studies indicating that the incubation approach generally yields the lowest efficiency. In contrast, physicochemical methods such as sonication and saponin or hypotonic dialysis can significantly enhance drug loading capacity, achieving superior loading efficiency (Fuhrmann et al., 2015; Kim et al., 2016). Consequently, optimizing and selecting high-efficiency loading strategies is crucial for realizing effective siRNA delivery.

Undoubtedly, continued advancements in drug loading technologies, EV engineering, and clinical validation will pave the way for widespread adoptions in treating a variety of diseases, including AKI.

### 4.7 Inorganic nanocarriers

Inorganic materials offer a highly adaptable foundation for constructing nanocarriers that allow precise control over both particle size and morphological characteristics. These substances display distinct advantages, such as outstanding biocompatibility, minimal immunogenic response, low toxicity, straightforward scalability, and convenient surface modification capabilities (Zhang et al., 2023).

Carbon nanotubes (CNTs) are cylindrical molecules composed of carbon atoms arranged in thin graphite sheets of condensed benzene rings, rolled into hollow cylinders (Moazzam et al., 2024). They have demonstrated effectiveness in siRNA delivery when functionalized using specific methods. Ammonia-functionalized single-walled carbon nanotubes (fCNTs) represent a unique class of fibrous macromolecules with highly favorable glomerular filtration and elimination properties, attributed to their large aspect ratio (McDevitt et al., 2007). A portion of the filtered fCNTs is reabsorbed at the PTC brush border and subsequently endocytosed (Ruggiero et al., 2010). This enables fCNTs to transport noncovalently bound siRNA to and within critical PTC physiological compartments, offering a promising approach for treating kidney-related pathologies (Alidori et al., 2013). Alidori et al. demonstrated the targeted delivery of siRNA against Trp53 and Mep1b to PTCs using a fCNT platform. This approach effectively prevented renal injury following a nephrotoxic insult, subsequently reducing fibrosis and immune cell infiltration. Notably, in a step toward clinical application, fCNTs were evaluated for the first time in nonhuman primates. The rapid, kidney-specific pharmacokinetic profile observed in primates was consistent with findings in mice, suggesting that this approach holds promise for human therapeutic use (Alidori et al., 2016).

As mentioned previously, inorganic NPs demonstrate therapeutic potential for AKI through their antioxidant properties and ultrasmall size. Despite challenges such as low targeting efficiency, insufficient long-term safety data, and difficulties in standardized production, these siRNA delivery systems offer unique advantages of stability, multifunctionality, and treatment precision that warrant further development.

## 5 Challenges and perspectives

siRNA therapy holds great promise as a treatment for AKI and its chronic progression. However, to fully harness the potential of siRNA therapeutics, there are still formidable challenges including identifying target genes, delivery systems, immune system clearance, and adverse off-target effects.

### 5.1 Target gene selection

Recently, remarkable advancements have been seen in omics technologies, particularly next-generation sequencing and mass spectrometry. Such progress has accelerated the evolution of omics research into quantitative and high-throughput paradigms. Multi-omics integration, which holistically combines genomics, proteomics, metabolomics, and transcriptomics, has become a pivotal approach for identifying disease-specific profiles, improving diagnostic accuracy, and promoting personalized therapeutic interventions (Ma et al., 2022). The synthesis and analysis of multi-omics data have revolutionized modern biology by uncovering sophisticated regulatory networks of genes and proteins that drive mechanistic pathways involved in both physiological and pathological processes throughout the progression of kidney injury, including initial AKI and its transition to chronic pathology. As a result, the discovery of genetically validated targets for RNAi therapeutics is expected to accelerate. Moreover, the application of advanced computational methods and artificial intelligence has further enhanced the analysis of multi-omics data, offering the potential for more precise and targeted therapeutic strategies to address disease progression (Rroji and Spasovski, 2024). For instance, Susztak et al. established a gene expression database derived from micro-dissected human kidney samples, which were separated into tubular and glomeruli. By conducting genome-wide association studies (GWAS), the team detected close to 250 genetic loci where variants correlate with renal function (Ko et al., 2017; Wuttke et al., 2019; Sullivan and Susztak, 2020). Guan et al. further performed computational integration of kidney function GWAS with methylation and expression quantitative trait loci, as well as human single-nucleus ATAC sequencing data. First, integration of GWAS with kidney methylation and expression quantitative trait loci (mQTL and eQTL) revealed that nucleotide variants in this region were associated with cytosine methylation levels and with the expression of dipeptidase 1 (DPEP1) and charged multivesicular body protein 1A (CHMP1A). Subsequently, statistical colocalization (Moloc analysis) indicated a high posterior probability (PP_abc = 0.92) that the eGFR GWAS, mQTL, and eQTL signals share common causal variants. To narrow down the likely causal variants, they utilized human kidney single-nucleus ATAC-seq, which identified 12 accessible chromatin regions in PTCs; conditional analysis implicated several of these peaks. Finally, CRISPR genome editing established the functional impact of these regions, demonstrating that deleting distinct peaks (e.g., peaks 8 and 9 for DPEP1 and peaks 8 and 12 for CHMP1A) directly and specifically altered the expression of each gene, thereby validating their roles as key effector genes in kidney disease. Further mechanistic studies revealed that both DPEP1 and CHMP1A are crucial regulators of ferroptosis. While Dpep1 altered iron import, Chmp1a interfered with iron export, indicating an important mechanistic convergence. Targeting these genes pharmacologically to modulate ferroptosis in PTCs could thus represent a promising therapeutic strategy for kidney disease patients (Guan et al., 2021).

However, it should be emphasized that potential target genes should be carefully selected, in different stages of AKI including both initiation and chronic progression, based on the following principles: (1) Target genes should play a significant role in the mechanisms underlying AKI; (2) Preferential expressed at the site of injured kidneys; (3) The location of target genes should be accessible by siRNA delivery systems; (4) Preferably a broad-spectrum of genes are applicable to multiple causes-induced AKI models. These principles not only guide the selection of target genes with broad applicability in the future, but also jointly define the safety and efficacy of treatment regimen.

The practical application of these principles can be illustrated by examining specific genes across different subtypes of AKI. The tumor suppressor gene p53 serves as a prime example of the specificity of cellular subtype. Molitoris et al. demonstrated that intravenous administration of p53-targeting siRNA at 4 h post-renal ischemia protected against apoptosis and preserved renal function. The siRNA was primarily taken up by PTCs within the kidney, indicating a targeted therapeutic effect (Molitoris et al., 2009). Furthermore, Yang et al. and Ying et al. showed that targeted deletion or acute inhibition of p53 from renal proximal tubules prevented interstitial fibrogenesis after acute renal ischemia injury in mice (Yang et al., 2010; Ying et al., 2014). Of note, the renal protective role of p53 ablation appears to be exclusive to the proximal tubules, as its deletion from other renal tubular segments did not translate to a protective phenotype (Zhang et al., 2014). This cell specific action makes p53 an excellent candidate for a therapy targeted to AKI, but it also necessitates delivery systems that can preferentially access PTCs to minimize off-target effects. Besides, the role of p53 in other clinically important forms of AKI, such as those associated with sepsis or contrast medium exposure, remains elusive. In contrast, as a widely expressed protease and key executioner of apoptosis, Caspase-3 is activated by diverse extrinsic and intrinsic death signals, serving as a common pathogenic mediator across different AKI etiologies and thereby fulfilling the criterion of a broad-spectrum target (Yang et al., 2014; Yang et al., 2011; Yang et al., 2018; Liu et al., 2025). However, their ubiquitous involvement in cell death pathways and normal physiological functions raises potential safety concerns (Eskandari and Eaves, 2022), illustrating the trade-off between efficacy and specificity.

All in all, a principle framework that integrates multi-omics insights and incorporates rigorous evaluation of canonical targets such as p53 and Caspase-3 will be essential to systematically identify optimal targets for siRNA-based therapies in AKI.

### 5.2 Targeted enrichment and internalization

Due to the diversity of cell types and the structural complexity of renal tissue, effectively enriching siRNAs in kidney tissue and ensuring their internalization into target cells are challenging (Ahn et al., 2023). The recent development of delivery technologies for siRNA therapeutics has brought hope to the treatment of AKI. Different strategies have been proposed to tackle the former, including adding kidney-selective ligands to the nanoparticles (Wischnjow et al., 2016). One recent, Vaidya et al. provided evidence of siRNA Selective Organ Targeted lipid nanoparticles achieving persistent extrahepatic gene silencing in the kidneys, lungs, and spleen (Vaidya et al., 2024). This is considered a promising method. Moreover, several methods have been developed to enhance the cellular internalization of siRNA drugs. These include optimizing chemical modifications to improve stability and interaction with cells (Guo et al., 2024), conjugating siRNA with cholesterol or other lipophilic molecules to increase membrane affinity (Li et al., 2024), and leveraging highly expressed receptors on target cells to promote uptake through receptor-mediated endocytosis (Tang et al., 2022b). Recently, stimulus-responsive nanoplatforms have emerged as a promising strategy to enhance cellular internalization. These nanoplatforms can be categorized into those triggered by endogenous stimuli—such as reactive oxygen species, pH, enzymes, or temperature and those activated by exogenous stimuli, including near-infrared light, ultrasound, or magnetic fields (Jiang and Zhang, 2023). A notable example is the pioneering “sonoporation” technique developed by Ishida, which transiently increases glomerular filtration barrier permeability to enhance tubular siRNA uptake. While promising, this approach requires further optimization to address its invasive nature before clinical translation (Ishida et al., 2016).

### 5.3 Efficient endosomal and lysosomal escape

Endosomal and lysosomal escape remains a significant bottleneck in the development of siRNA therapeutics. Studies have shown that only a small fraction of internalized siRNA molecules successfully escape into the cytosol, while the majority are either degraded by lysosomal enzymes or recycled out of the cell (Dowdy, 2017). To date, attempts to enhance endosomal escape using modified pH-sensitive materials, ion-penetrating agents, chloroquine-like lysosomotropic compounds, or pore-forming peptides have struggled to balance increased escape efficiency with cytotoxicity (Guo et al., 2024). Recently, Qiu et al. demonstrated a novel approach involving endoplasmic reticulum (ER) membrane decoration on nanoparticles. This method effectively facilitates siRNA transport through the endosome-Golgi-ER pathway, avoiding lysosomal degradation and enhancing the gene-silencing effects of siRNA (Qiu et al., 2019). This technique holds promise as a future strategy for nucleic acid drug delivery. Additionally, leveraging natural delivery systems, such as extracellular vesicles (including exosomes), or components derived from these vesicles, offers another promising avenue. Indeed, research has shown that RNA-loaded REVs exhibit strong therapeutic potential in treating AKI and mitigating its chronic progression (Tang et al., 2021). Moving forward, unraveling the precise mechanisms of endosomal release in RNA therapies will be crucial. This deeper under-standing could drive the development of more effective delivery methods or advanced carrier systems.

### 5.4 Safety and tolerability

Good biocompatibility, biodegradability, and low toxicity are essential characteristics of an effective targeted drug delivery vector. Because of technological advancements, new materials and chemical modifications have been widely adopted to enhance siRNA stability, minimize or eliminate immunogenicity and toxicity (including off-target effects), whereas challenges still persist (Guo et al., 2024). For example, chemically synthesized carrier systems may induce apoptosis and inflammation *in vivo*, while modifications aimed at improving stability and specificity can enhance delivery, they may also reduce silencing efficacy or lead to unforeseen adverse effects (Guo et al., 2024). Future re-search should focus on addressing these fundamental issues. Recently, combination therapies have also been explored, including dual-targeting approaches and the co-administration of other drug types (such as small molecules or biologics) (Li et al., 2021). This strategy utilizes siRNA at lower concentrations to reduce off-target effects without compromising silencing potency. Such approaches have shown significant promise, particularly in treating cancer (Liu et al., 2014) and hepatitis B virus infections (Tang Y. et al., 2022). Moreover, while non-primate models remain a staple in preclinical RNA drug research, they often fail to replicate the full genomic overlap with humans, limiting the predictability of pharmacodynamic effects. Expanding the use of non-human primate models, or even disease-related organoids, may offer a more accurate alternative (Guo et al., 2024). Lastly, delivery systems must be designed to ensure ease of production, stringent quality control, and efficient transport, all crucial for large-scale clinical applications (Humphreys et al., 2022).

## 6 Conclusion

The treatment of siRNA is highly effective, in terms of timely and temporarily inhibiting detrimental genes in AKI, and significantly improving short and long-term outcomes. Selecting target genes and cells alike at different stages of AKI can be achieved by analyzing multi-omics data from kidneys and integrating comprehensive understanding of gene regulation. Additionally, developing effective drug delivery systems and optimizing kidney/cell-targeting strategies are needed to overcome extra-cellular and intracellular barriers. With the continued progress of knowledges and the rapid advancement of technologies, siRNA therapy is encouragingly poised to success in the near future.
